# Effects of known functional alleles of agronomically important genes on yield-related traits estimated by historical rice breeding data in Japan

**DOI:** 10.1270/jsbbs.25062

**Published:** 2026-06-11

**Authors:** Koki Chigira, Eiji Yamamoto, Akitoshi Goto, Tomohito Ikegaya, Nobuhiro Suzuki, Yoshihiro Kawahara, Masanori Yamasaki, Kazuhiko Sugimoto, Kiyosumi Hori

**Affiliations:** 1 Institute of Crop Science, National Agriculture and Food Research Organization, 2-1-2 Kannondai, Tsukuba, Ibaraki 305-8518, Japan; 2 Graduate School of Agriculture and Life Science, The University of Tokyo, 1-1-1 Yayoi, Bunkyo, Tokyo 113-8657, Japan; 3 Research Center for Advanced Analysis, National Agriculture and Food Research Organization, Tsukuba, Ibaraki 305-8518, Japan; 4 Graduate School of Science and Technology, Niigata University, 8050 Ikarashi 2-no-cho, Nishi-Ku, Niigata City, Niigata 950-2181, Japan

**Keywords:** rice, yield, natural variation, modeling, historical data

## Abstract

Utilizing genetic information is a promising strategy to accelerate crop breeding. The identification of many causative genes in rice, even for polygenic traits, enables us to take on the challenge of predicting yield-related traits by using allele information. However, phenotyping has been a bottleneck in such analyses. Historical breeding data constitute a valuable source of yield-related phenotypic data. Here, we used a subset of 19,218 records drawn from 225,163 records in the rice historical phenotype dataset maintained by the National Agriculture and Food Research Organization. We evaluated the heritability of seven yield-related traits explained by 26 agronomically important genes and the accuracies of the models’ predictions. These genes constituted a portion of the total genetic effect, but the effects of the genetic background were also significant. In some traits, the model based on the agronomically important genes proved more accurate than that based on whole-genome polymorphisms. We also estimated the effects of genes that contribute to yield. This paper presents the possibilities and challenges of utilizing historical breeding data, and important information on how to utilize allele information in future breeding.

## Introduction

Global climate change is accelerating, increasing the demand for accelerating crop breeding to adapt to changing environments. Rapid and cost-effective breeding of crops is essential. Advances in next-generation sequencing technology have made it easier to obtain genotype data. However, phenotyping remains one of the most important limiting factors in crop breeding (Chawade *et al.* 2019). Therefore, a lot of effort has been devoted to replacing phenotypic selection with genotypic selection (Lamichhane and Thapa 2022).

The concept of marker-assisted selection (MAS) was first proposed in the 1980s with the development of DNA markers (Dudley 1993, Paterson *et al.* 1988). MAS is inseparable from the concept of quantitative trait loci (QTLs). In rice (*Oryza sativa* L.), many QTL analyses have been conducted to identify QTLs and genes responsible for various agronomic traits such as heading date, grain yield, disease resistance, and abiotic stress tolerance. Among such genes, *Hd1* and *Ghd7* are related to heading date, *SD1* and *NAL1* to plant architecture, *Gn1a* and *APO1* to grain number, and *GS3* and *GS5* to grain size (Ashikari *et al.* 2005, Fan *et al.* 2006, Li *et al.* 2011, Ookawa *et al.* 2010, Sasaki *et al.* 2002, Takai *et al.* 2013, Xue *et al.* 2008, Yano *et al.* 2000). MAS is based on genotypes of markers that are linked to such responsible genes or QTLs. MAS has been applied to a wide range of breeding schemes, including backcrossing, pyramiding, pedigree breeding, and recurrent selection (Eathington and Crosbie 2007, Ribaut and Ragot 2006). It has allowed pyramiding multiple monogenic traits (e.g., disease resistance) or QTLs for a single target trait (e.g., drought tolerance) (Xu and Crouch 2008). However, its effect on rice breeding was lower than expected (Collard and Mackill 2008), generally because many agronomic traits, including yield-related traits, are quantitatively inherited and are influenced by many unknown genes with small effects (Bernardo 2008, Spindel *et al.* 2015).

Genomic selection (GS) was proposed as an alternative means to improve quantitative traits in crop species (Crossa *et al.* 2017). GS is based on genomic prediction models that use genome-wide DNA markers. Unlike MAS, GS does not consider whether DNA markers are linked to QTLs. It has been successful in improving yields of maize and wheat cultivars (Atanda *et al.* 2021, Dreisigacker *et al.* 2021, He *et al.* 2016). However, only its theoretical application to rice breeding has been reported (Spindel *et al.* 2015).

Among various crop species, rice is the subject of many advanced genomics and genetic research projects, and many genes responsible for phenotypic differences among cultivars have been identified so far. For example, natural variations of more than 30 genes can affect yield through sink or source capacity, and those of more than 10 genes can affect yield through heading date (Hori *et al.* 2016, Ueda *et al.* 2025). The Rice Annotation Project Database (RAP-DB) listed 365 agronomically important rice genes and 762 known functional alleles/mutations of them in 2022 (Kawahara *et al.* 2022), and the list has been updated continuously since (https://doi.org/10.64898/2026.01.16.699882). This has made it possible to construct prediction models based on known functional alleles of agronomically important genes. Spindel *et al.* (2016) reported that accuracies of prediction of heading date, plant height, and yield improved in genomic models when DNA markers associated with a trait were added as fixed effects. Wei *et al.* (2021) summarized eight genome-wide association studies and quantified the effects of alleles of 69 quantitative trait genes on heading date, plant height, and grain number and size. Kawakita *et al.* (2024) reported a high-accuracy model for the prediction of heading date using genotypes of 11 flowering genes and a phenology model. Since advancements in next-generation sequencing technology have made it possible to obtain genomic information from many individuals, the primary obstacle in genomic prediction research is now the acquisition of extensive phenotypic data.

Historical breeding data provide a good phenotype resource for constructing genomic prediction models because they contain a large amount of phenotype scores from multiple cultivars and years. Such data proved to be useful for predicting grain yield from genetic and environmental data in wheat, maize, and soybean (de los Campos *et al.* 2020, Jarquin *et al.* 2016, Khaki and Wang 2019). Promising Japanese rice lines are evaluated for yield and other characteristics at multiple public agricultural research institutes before registration as cultivars, and a database of their phenotypes is maintained by the National Agriculture and Food Research Organization (NARO) (Matsushita *et al.* 2024, Ohsumi *et al.* 2022). This NARO rice historical phenotype dataset is now being used in rice modeling studies (Shimono *et al.* 2023, Taniguchi *et al.* 2025).

Here, to quantify effects of known genes associated with grain yield and related traits under diverse genetic and environmental backgrounds, we built prediction models using the latest NARO phenotype dataset and known functional alleles of agronomically important genes as genotypic data. We posed three questions: (1) How much can the heritability of yield-related traits be explained by the genes? (2) What is the prediction accuracy? (3) How much influence do individual genes have on yield-related traits?

## Materials and Methods

### Extraction of phenotypic data

We extracted a subset of phenotypic data from the latest NARO phenotype dataset. Whole-genome sequence data are necessary to investigate associations between traits and genotypes, but the dataset contains limited numbers of fully sequenced cultivars. And as breeders generally grow cultivars that are suitable for their region for evaluation, there are strong biases in combinations of cultivars and locations. For example, if one cultivar is grown at high latitudes, while another is grown only at low latitudes, it is difficult to calculate whether variations in phenotypic traits are due to differences in cultivars or in locations. There are also large differences in environmental conditions and crop management among locations. To reduce these biases, we extracted a subset of data as follows:

(1) We removed records that describe direct seeding, upland cultivation, or extremely high fertilizer rates.

(2) We extracted trials (combinations of year and location) with a record of the common tester ‘Koshihikari’ (cultivar ID L029).

(3) We selected records so that all records included in the same trial had a single cultivation condition (seeding day and fertilization).

(4) In grain yield, when a given cultivar appears with multiple records within a trial, the median value was used as its representative value.

(5) We extracted cultivars with available genomic data.

(6) We extracted trials with five or more cultivars.

(7) We extracted cultivars whose yields were recorded in five or more trials.

(8) We extracted locations used in three or more trials.

(9) We removed records missing sowing date, heading date, or grain yield.

(10) We removed records that appear to have recording errors (ex. days to heading = 0).

The 153 cultivars in the subset are listed in Supplemental Table 1. Connectivity indexes of the extracted subset were calculated as described (de los Campos *et al.* 2020). The phenotypic and genotypic data are not publicly available due to intellectual property issues. However, the data that support this study are available upon request.

### Genotype data

We reanalyzed short-read Illumina sequencing data archived in DRA000307, DRA000897, DRA000927, and DRA007273 and used them for genotyping (Hamazaki *et al.* 2020, Jarquin *et al.* 2020, Yabe *et al.* 2018). We also used data archived in DRA021524, DRA021525, DRA021526, DRA021527, and DRA021528 for genotyping. From raw reads, low-quality bases and adaptor sequence were trimmed in Trimmomatic software version 0.40 (Bolger *et al.* 2014). Trimmed reads were mapped to the ‘Nipponbare’ reference sequence, IRGSP-1.0 in BWA software version 0.7.17 (Li and Durbin 2010). Mapped reads were sorted, and PCR duplications were marked in Picard software version 3.4.0 (Broad Institute, https://broadinstitute.github.io/picard/). SNPs and indels in each cultivar were genotyped in GATK software version 4.6.2.0 (Van der Auwera and O’Connor 2020). The parameters used in this pipeline were the same as those in the analysis workflow for genome-wide variations in TASUKE+ for RAP-DB v. 2.0 (https://rapdb.dna.affrc.go.jp/genome-wide_variations/genome-wide_var_workflow_v2.html) (Kumagai *et al.* 2019). SNPs and indels with missing rate >0.1 or minor allele frequency <0.025 or multiple alternative alleles were excluded from the resultant variant call format (VCF) file, and genotypes were imputed in Beagle software version 4.1 (Browning and Browning 2007). Finally, 1,470,254 polymorphisms without missing records were generated.

### Tabulating known functional alleles of agronomically important genes

We extracted DNA polymorphisms of a set of yield-related genes associated with heading date, plant type, grain size, and grain quality from VCF files of the extracted 153 cultivars. The genes were collected from the table of agronomically important genes, and their functional alleles were collected from the table of known functional alleles and mutations in RAP-DB (https://rapdb.dna.affrc.go.jp/). Different alleles considered to have the same degree of effect were integrated into the one category. Finally, 56 known functional alleles for 26 agronomically important genes were determined (Supplemental Table 2). Alternative alleles were named according to their functions (Supplemental Table 2).

### Prediction models

To quantify the genetic effect explained by natural variations in major genes for seven traits, we fitted the phenotypic data to four models in the BGLR package version 1.1.4 using Markov chain Monte Carlo methods (Pérez and de los Campos 2014). We set number of iterations (nIter) = 12000, burn-in period (burnIn) = 2000, and sampling interval (thin) = 10. Model convergence was checked through visual inspection of the trace plots of the parameters. The seven traits were days to heading (DTH), culm length (CL), panicle length (PL), panicle number (PN), total dry weight (TOTALWT), thousand-grain weight (TGW), and grain yield (YIELD). TGW and YIELD were measured using brown rice after sieving. All models included an intercept (μ) plus the normal, independent and identically distributed (NIID) random effects of year ($Y_{i}$), location ($L_{j}$), year × location (${YL}_{ij}$), and genetic factor (by type of model):

Cultivar (CV) model: $y_{ijk}=\mu +Y_{i}+L_{j}+{YL}_{ij}+V_{k}+\varepsilon_{ijk}$

Known-gene (KG) model: $y_{ijk}=\mu +Y_{i}+L_{j}+{YL}_{ij}+{\sum_{n}^{N}\alpha }_{n}x_{kn}+\varepsilon_{ijk}$

Whole-genome (WG) model: $y_{ijk}=\mu +Y_{i}+L_{j}+{YL}_{ij}+G_{k}+\varepsilon_{ijk}$

Known-gene + background (KG + BG) model: $y_{ijk}=\mu +Y_{i}+L_{j}+{YL}_{ij}+{\sum_{n}^{N}\alpha }_{n}x_{kn}+{G^{\prime }}_{k}+\varepsilon_{ijk}$

Where $V_{k}$ is a random cultivar effect that is also NIID; $\alpha_{n}$ represents the effect of know functional allele *n*, and $x_{kn}$ denotes the coded variable whether cultivar *k* possesses the know function allele *n* (Supplemental Table 3). $G_{k}$ is the genetic effect with a covariance matrix proportional to a genomic relationship matrix constructed from 1,470,254 whole-genome polymorphisms as in de los Campos *et al.* (2020). The genomic relationship matrix was calculated by dividing the cross product of the matrix of genotypes of the polymorphisms by the number of SNPs; ${G^{\prime }}_{k}$ is the genetic effect calculated similarly to $G_{k}$, but constructed from 496,138 polymorphisms where $r^{2}$ values, which represent linkage disequilibrium with each known functional allele, were all lower than 0.6; and $\varepsilon_{ijk}$ is residuals. The CV model includes the random effect of cultivar. The KG model replaces that with a term for the known functional alleles of agronomically important genes. Different haplotypes that have the same phenotypic effect were treated as the same allele. To evaluate prediction accuracies of the KG model, we constructed a control model using WG polymorphisms as genetic effects. The WG model is based on the same concept as the genomic prediction model used in GS. KG + BG model was constructed to evaluate the polygenic effects ignored in KG model.

We calculated the proportion of phenotypic variance explained (PVE) by year, location, year × location, and genetic factor (cultivars in CV model, known genes in KG model). PVEs were calculated by dividing variances of each component by sum of variances. The variances were extracted from the BGLR output file “varE.dat” for residuals and “ETA_[variable name]_varB.dat” for other effects. PVE by cultivar indicates the total genetic effect or heritability of the traits. PVE by known functional allele indicates how well the genes explain the total genetic effect. We examined two regression methods, Bayesian ridge regression (BRR) and Bayes B. Their prior distributions were the defaults of BGLR package described in Pérez and de los Campos (2014).

We used a 10-fold cross-validation with cultivars assigned to folds to assess prediction accuracy. The detailed scheme is described below. The 153 cultivars were randomly divided into 10 groups of 15 or 16 cultivars each. Models were constructed using a dataset excluding records with cultivars belonging to one of these groups, and the traits of test data were estimated. The correlation coefficient between the estimated values and the true trait values in the test data was calculated. This was performed for all 10 groups, and the average correlation coefficient was calculated. This process was repeated 10 times with different random seeds, and the mean and standard deviation of the correlation coefficients were shown.

## Results

### Summary of extracted phenotypic data

The latest version of the NARO rice historical phenotype dataset contained 225,163 records including 5,236 cultivars in 111 locations with 3,680 trials spanning 43 years from 1980 to 2022 (Table 1). Among the data, we selected 19,218 records including 153 cultivars in 81 locations with 2,176 trials spanning 43 years as a subset for our research. The locations of the extracted phenotypic data were evenly distributed in Japan (Fig. 1). The northern limit was 38.8°N, and the southern limit was 31.5°N (Fig. 1).

To quantify the genetic effects accounting for phenotypic variance accurately, it is desirable to include many common cultivars between trials. When we use the connectivity index ($\bar{T_{\left(r\right)}}$) described in de los Campos *et al.* (2020), $\bar{T_{\left(2\right)}}=1564.4,\bar{T_{\left(3\right)}}=832.8,\bar{T_{\left(4\right)}}=362.5,\bar{T_{\left(5\right)}}=137.9$, in the subset (Supplemental Fig. 1). This means that each trial was connected to 833 other trials (out of 2,175 trials) on average through at least three cultivars, and to 138 other trials on average through at least five cultivars. Although records for some major cultivars were available in many years, records for other cultivars were available for only a short period (Supplemental Fig. 2).

Phenotypic values of all seven traits had a normal distribution (Fig. 2). PN, TOTALWT and YIELD were slightly but positively correlated with DTH (Supplemental Fig. 3).

### Genetic effect explained by known functional alleles of agronomically important genes

We selected 26 genes whose functional alleles affecting traits are reported and determined 56 known functional alleles among the 153 cultivars (Supplemental Table 2). Four genes had three known functional alleles, and the rest had two. The 26 genes comprise *Hd1*, *Ghd7*, *DTH8*, *Hd6*, *Hd16*, *Hd17*, *Hd18*, *Ehd1*, *OsMADS51*, *DTH2*, *OsHESO1*, and *OsGATA28* for heading date; *SD1*, *OsTB1*, *NAL1*, *HTD1*, and *OsSPY* for plant architecture; *Gn1a*, *APO1*, and *DEP1* for panicle architecture; *GS3*, *GS5*, *GW6a*, *GW7*, *GW8* for grain size; and *Wx* for grain quality.

Regarding the two regression methods, BRR and Bayes B, showed largely the same results (Supplemental Fig. 4). We described the results of BRR below. In the CV model for DTH, PVE by location was the largest among all factors, at 55.0%, genetic effect was 27.4%, and the error variance was 3.8% (Fig. 3). In the KG model for DTH, genetic effect was 24.2%. In the KG + BG model for DTH, genetic effect was 23.0%, with 13.0% due to known genes and 10.0% due to genetic background other than the known genes. In the CV model for CL, genetic effect was largest among all traits, at 56.7%. However, the genetic effect of KG model was 38.8%, and the genetic effect due to known genes in the KG + BG model was 22.2%. PVEs for PL resembled those for CL in all models. PVEs for PN and TOTALWT resembled those for DTH, with larger error variances. In particular, the genetic effect of KG model and the genetic effect due to known genes in the KG + BG model for PN was estimated lower than those for DTH and TOTALWT. PVEs for TGW resembled those for CL, but the genetic effect due to known genes was relatively small. For YIELD, the genetic effect of CV model was 12.5%. The genetic effect of KG model was 8.0%. The genetic effect of KG + BG model was 13.0%, with 3.4% due to known genes and 9.6% due to genetic background.

### Prediction accuracy of KG model

The prediction accuracies of the KG model and KG + BG model were higher than those of the WG model for DTH, CL, and TOTALWT (Fig. 4). For PL, PN, TGW, and YIELD, KG + BG model and WG model showed almost same prediction accuracies, but those of KG model were lower than them.

### Effects of each known functional allele

The KG + BG model estimated the effects of each known functional allele. *Hd1* had the largest estimated effect on DTH (–23.8 days by “Hd1_early” allele), followed by *Hd16* (–18.1 days by “Hd16_early” allele) (Fig. 5). The “HESO1_late” allele delayed heading by 3.6 days, “Hd6_late” by 8.6 days, “Hd17_late” by 9.3 days, and “Hd18_late” by 4.7 days (Fig. 5). The “SD1_short” allele had estimated effects on CL and PL of –16.8 cm and –0.5 cm, respectively (Fig. 5). “Hd1_early” and “Hd16 early” had estimated effects on CL of –14.5 cm and –7.2 cm, respectively. The effects on TOTALWT resembled those on DTH.

An allele associated with grain size, “GS3_long”, had the largest estimated effect on TGW. Regarding YIELD, no genes except Hd1 showed a clear effect (Fig. 5). Alleles of “SPY_heavy”, “DEP1_dense”, and “GATA28_late”, which are associated with panicle-weight-type plant architecture, slightly reduced YIELD. “TB1_C117” and “NAL1_thin” had estimated effects on PN of –25.5 m^–2^ and 47.1 m^–2^, respectively. High-yielding staple rice cultivars released recently lacked some alleles associated with high yield, such as “Gn1a_more”, “HTD1_more”, and “APO1_large” (Table 2).

## Discussion

### Effects of agronomically important genes on the yield-related traits

We revealed the PVE among seven traits associated with yield by genetic factors and by environmental factors (year and location). Then we showed the extent to which agronomically important genes can explain the total genetic effect on the traits (Fig. 3). In DTH, the genetic effect of KG model explained the heritability well. DTH is an oligogenic trait rather than a polygenic trait and is easily observed, so causal genes and the interactions among them are relatively well understood (Hori *et al.* 2016). DTH can be largely estimated using haplotypes of known genes (Chen *et al.* 2020, Kawakita *et al.* 2024). Our results suggest that earlier models are also applicable to historical breeding data. However, For the other six traits, the total genetic effect could not be fully explained by known genes alone. It could be explained by both known genes and unknown polygenic effects shown in the KG + BG model. In addition, known gene effects estimated in the KG model were over-estimated.

We also visualized the involvement of individual genes in the model (Fig. 5). Among the major genes, the loss-of-function allele of *Hd1* had an estimated DTH effect of –24 days relative to reference allele. In a QTL analysis of *Hd1*, it had an estimated effect of –12 days in the ‘Koshihikari’ background (Takeuchi *et al.* 2008), and –29 days in the ‘Nipponbare’ background (Yano *et al.* 1997). Therefore, the *Hd1* allele effects obtained in this study are reasonable values. Similarly, effects of alleles in *Hd6*, *Hd16*, *Hd17*, and *Hd18* were reasonable compared to previous QTL studies (Hori *et al.* 2013, Matsubara *et al.* 2012, Shibaya *et al.* 2016, Takahashi *et al.* 2001). However, the coefficient for the late-heading allele of *Ghd7* was not consistent with observations in previous studies. This may be due to some gene × gene interactions that were not considered in our models because *Ghd7* lies the upstream in the network controlling heading date and interacts with many genes (Matsubara and Yano 2018).

The percentages of genetic effects of CL, PL and TGW were high in the CV model, but they were less explained by known functional alleles in the KG + BG model (Fig. 3). This suggests the existence of many unknown genes associated with CL, PL and TGW. TOTALWT is a complex trait, but the effect of known functional alleles was large, likely owing to the positive correlation between DTH and TOTALWT (Supplemental Fig. 3).

The total genetic effect for YIELD was the lowest among the seven traits (Fig. 3). This indicates that grain yield was strongly affected by differences in cultivation conditions among locations and by large random errors at each location. The PVE of known genes was even lower, and no individual known genes were detected to significantly affect YIELD, except for the “Hd1_early” allele, which had a negative effect on YIELD. This is consistent with the grain yield being a typical polygenic trait.

### Quality of the extracted phenotypic data

The database of Japanese rice cultivation trials contains yield data from enormous numbers of environments and cultivars, which is difficult to obtain in routine experiments. This suited our goal of evaluating the effects of known functional alleles on rice yield under various environments. However, data were collected not for the purpose of modeling but for evaluating performance before the registration of new cultivars, and many lines were recorded in at most a few locations and years, making their data unsuitable for modeling yields using genotypes and environments. In addition, the mesh size of the sieve used in the selection of brown rice might vary between trials, and the impact of this on YIELD and TGW was unknown.

‘Koshihikari’ is very popular and widely grown in Japan (Kobayashi *et al.* 2018). It has been used as a control cultivar in many trials to evaluate the performance of other cultivars. However, data from trials in northern and southwestern Japan could not be used, because ‘Koshihikari’ is not grown in those regions. The genetic bias due to this omission could influence our estimation of known genes’ effects.

In addition, the use of data from trials with only one cultivar in common is insufficient for building models with high accuracy. If such data were used, it is difficult to estimate the error variance of environmental effects. de los Campos *et al.* (2020) evaluated the degree of connectivity among the data using a connectivity index. The connectivity indexes considering connections of three to five cultivars in our dataset were clearly inferior to those in de los Campos *et al.* (2020). This is apparently because many cultivars in our dataset were evaluated only during a specific period before they had been registered, and only in a specific region where they were going to be grown. This is an unavoidable characteristic of the NARO phenotype dataset. Although it would have been possible to increase the connectivity index by thinning down the number of cultivars, this would have reduced genetic diversity. Our dataset strikes a balance.

### Potential of the gene information for selection in breeding

The KG model and KG + BG model predicted DTH, CL, and TOTALWT at a higher accuracy than the WG model (Fig. 4). An advantage of the WG model is that it can consider unknown genetic effects. On the other hand, the KG model can avoid the noise generated by incorporating variables unrelated to the phenotypic variation. The KG + BG model combines the features of both models. For these traits, it is likely that the advantages of the KG model were larger. In the variance component analysis, the PVE by genetic effects in the KG model was only about two-thirds of that in the CV model for CL (Fig. 3). However, in the genomic prediction, the KG model had higher prediction accuracy than the WG model (Fig. 4), likely because *SD1* is the key gene for CL in Japanese rice cultivars. In most modern Japanese rice cultivars, the semi-dwarfing allele of *SD1* comes from the *indica* cultivar ‘IR8’. Most Japanese staple rice cultivars are classified into the *japonica* subspecies, but cultivars with the semi-dwarfing allele have significant quantities of *indica* chromosome segments, with many polymorphisms, in the *japonica* background. Therefore, the low accuracy of the WG model at predicting CL may be due to the noise of *indica* polymorphisms. Bernardo (2014) concluded that when each major gene accounts for ≥10% of genetic variance, all of them should be fitted as having fixed effects in the genomic prediction model. This may be the case for *SD1* in the prediction of culm length.

The WG model and the KG + BG model predicted YIELD with higher accuracy than the KG model (Fig. 4). These results suggest that GS is a more promising tool than MAS for improving yield. On the other hand, high-yielding staple rice cultivars released recently in Japan lack some alleles that positive effects on yield have reported in previous studies (Table 2). Considering these circumstances, it is thought that yield improvement is being achieved by utilizing unknown polygenic effects rather than by accumulating known functional alleles. Our results support this approach. Furthermore, alleles for panicle-weight-type architecture had almost no effect on yield, or even a negative effect in our results (Fig. 5). However, panicle-weight-type was considered to be an ideal plant architecture for high-yielding cultivars in the Philippines and China (Peng *et al.* 2008, Qian *et al.* 2016). This maybe owing to differences in genetic background, but there may have not been enough attempts at breeding such cultivars in Japan. The accumulation of panicle-weight-type alleles could be a future strategy for increasing yield. On the other hand, some alleles that increase grain yield reduce grain quality. Only promising lines are recorded in the NARO phenotype dataset, and it is not possible to know the phenotypes and genotypes of eliminated lines. Sharing data on such failed experiences with breeders can make theoretical studies more meaningful for breeding.

The prediction accuracy of our models was based only on historical breeding data from multiple trials. When GS is applied in real breeding programs, training populations are likely to have more similar genetic backgrounds and to have been phenotyped in more uniform environments. In such a case, whole-genome models that can take into account unknown genetic factors would be more powerful than predictions based only on known genes. However, our results show that a dataset containing multiple environments and more diverse cultivars can be used to construct models with a certain level of prediction accuracy by using the causative genes behind the traits. Identifying causative genes of natural variations is still meaningful, not only academically but also for further practical breeding.

We extracted phenotypic data from the NARO rice historical phenotype dataset, balancing genetic diversity and connectivity among the data, and used the data to build models to predict seven traits associated with yield. We showed the effects of known functional alleles on yield-related traits, although there were challenges in utilizing the database with the models. Models using known functional alleles had higher accuracy at predicting some traits than models using whole-genome polymorphisms. This suggests that identifying the genes responsible for natural variation is still important for further practical breeding, as well as for understanding the genetic basis controlling agronomic traits.

## Author Contribution Statement

KC, AG, KS and KH designed this study. KC and EY performed modeling. AG, TI and NS contributed to make and update the database of Japanese rice cultivation trials. AG and MY contributed to get whole-genome resequencing data used in this study. YK provided basic data for determining the alleles of known genes. KC, EY and KH wrote the manuscript. All authors read and approved of the final manuscript.

## Supplementary Material

Supplemental Figures

Supplemental Tables

## Figures and Tables

**Fig. 1.**
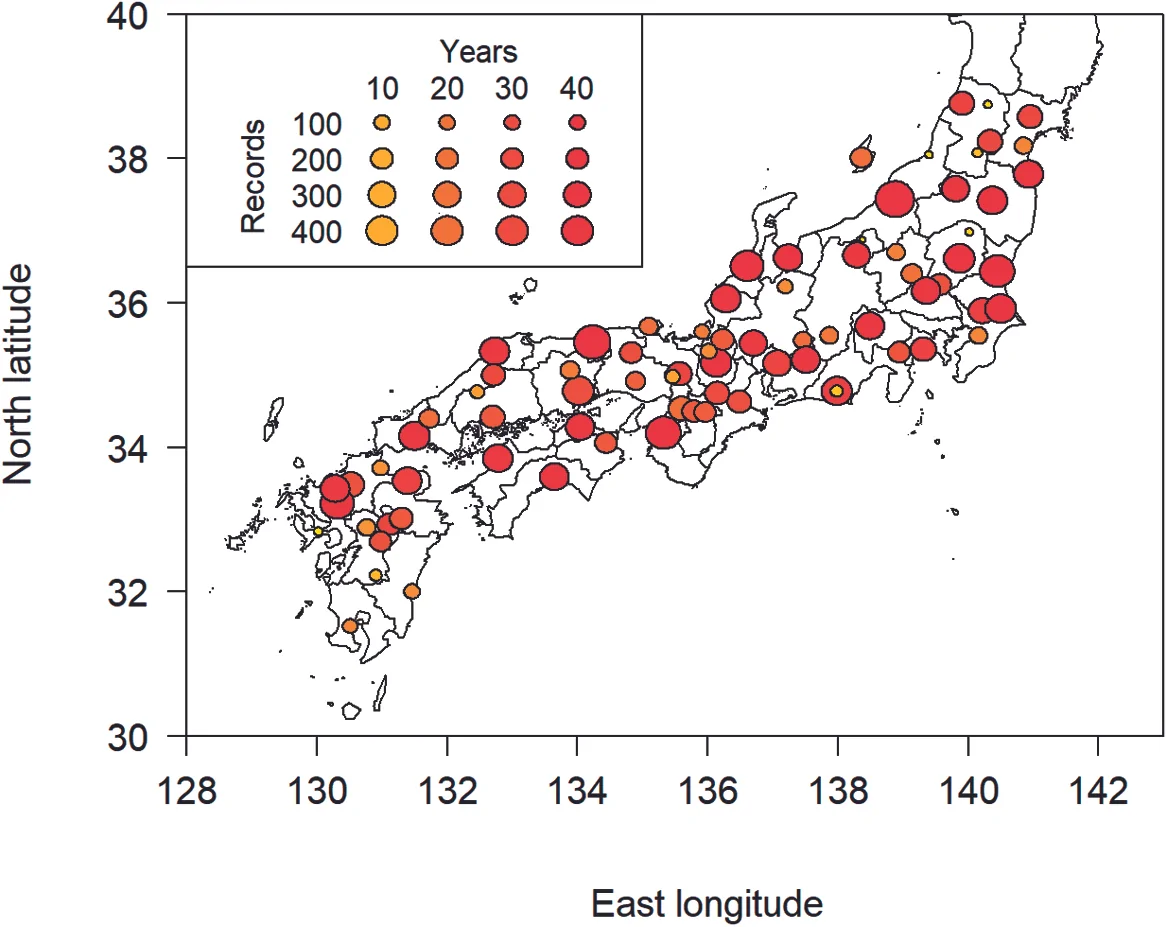
Locations where phenotypic data in the dataset were collected.

**Fig. 2.**
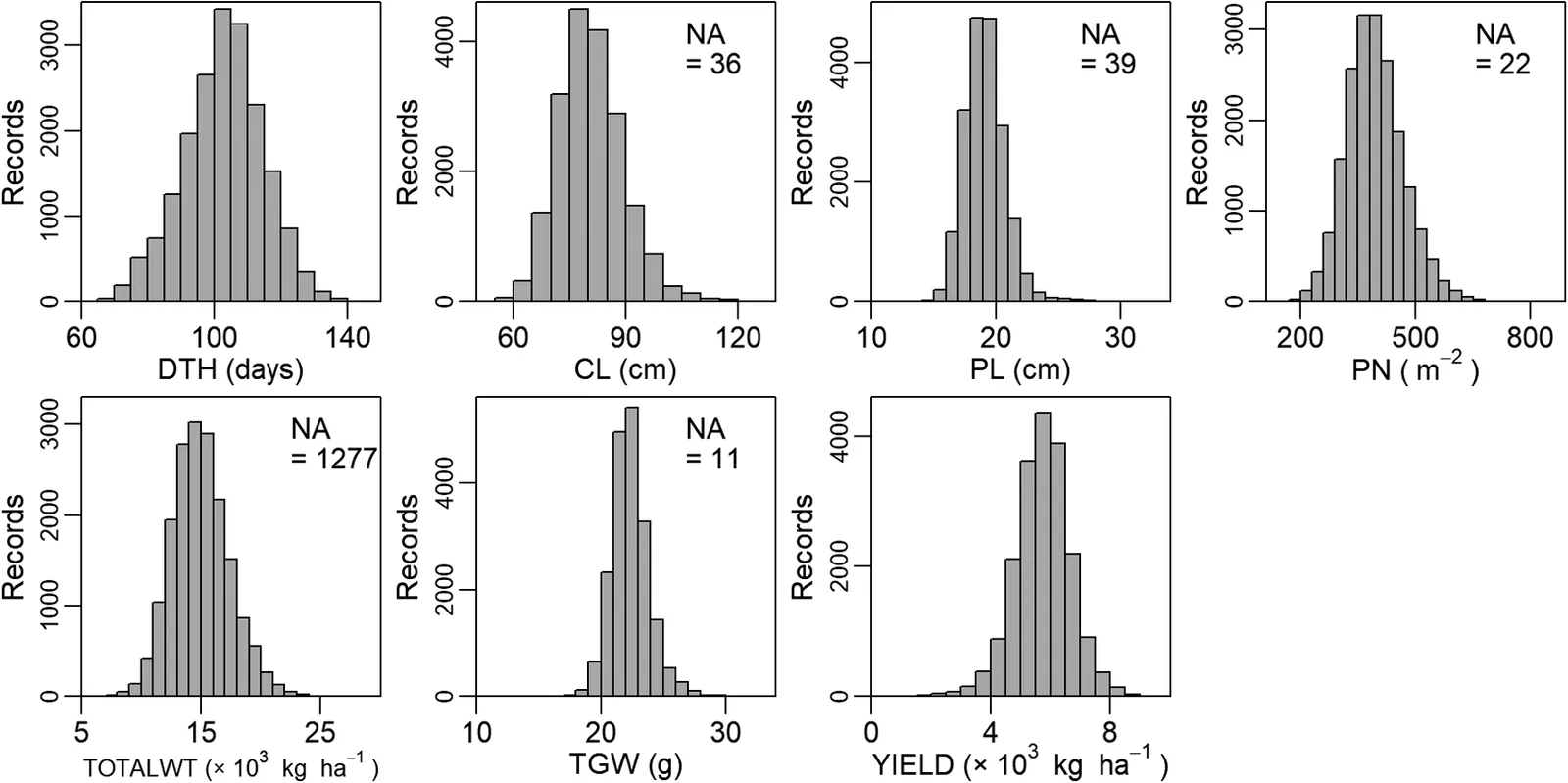
Histogram of phenotypic values of seven traits used in this study. The NA numbers indicate the number of trials in which the trait value was missing.

**Fig. 3.**
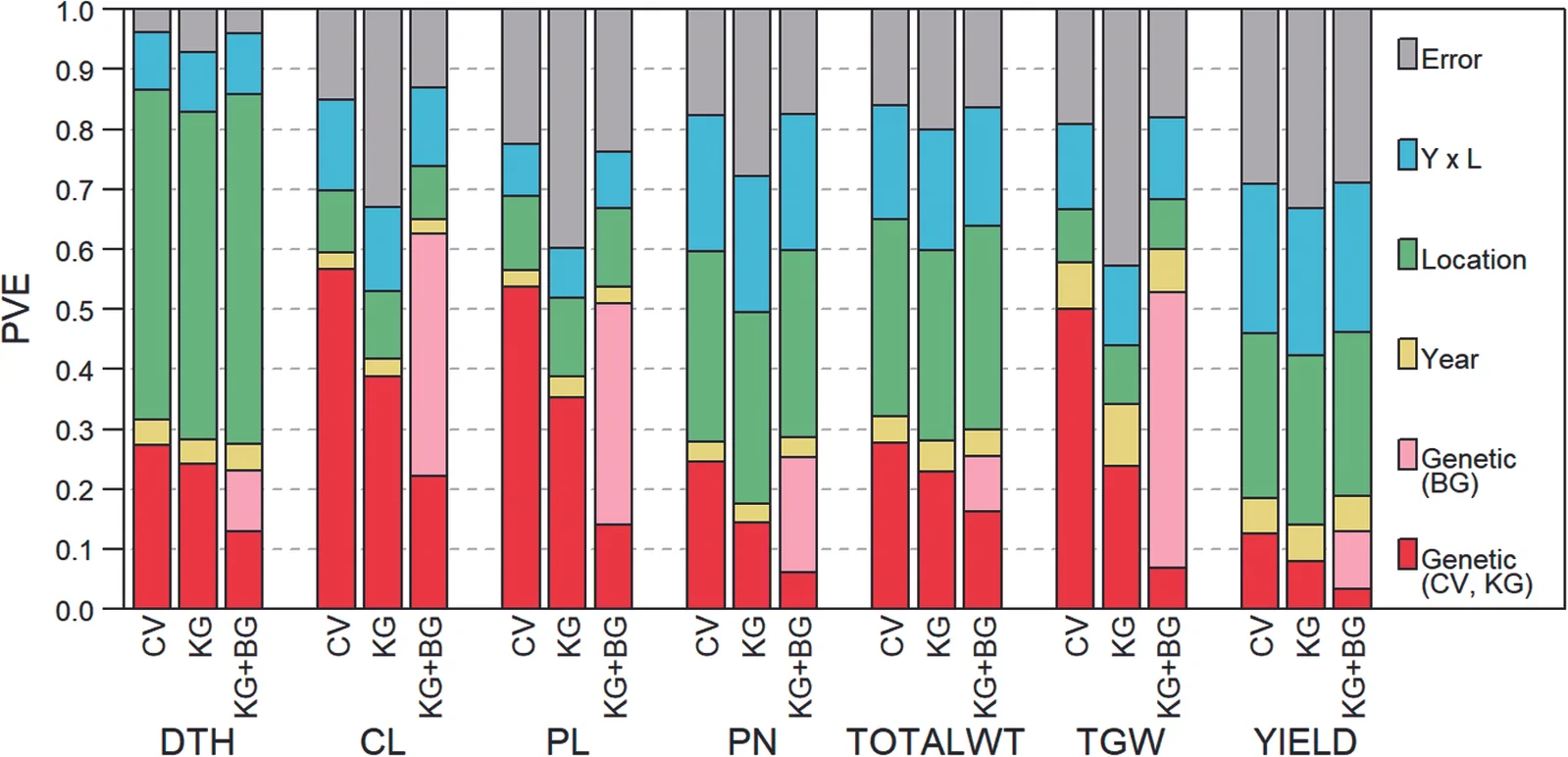
PVE by environmental and genetic factors in each trait calculated by CV (cultivar), KG (known-gene) models, and KG + BG (known-genes + background) models. The effects were estimated using BRR.

**Fig. 4.**
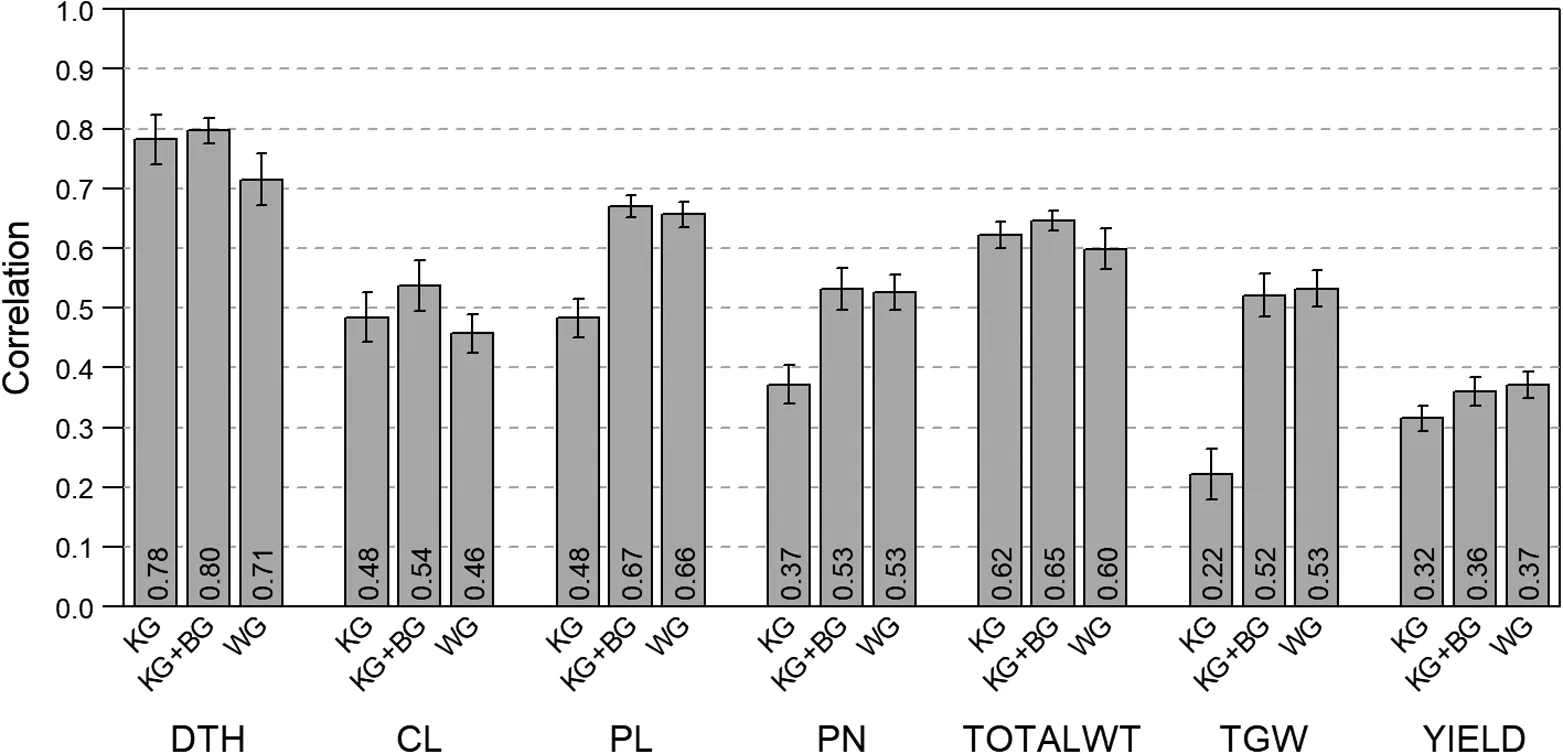
Prediction accuracy validated by means of coefficients of correlations between predicted and real values in test populations. Error bars indicate standard deviation.

**Fig. 5.**
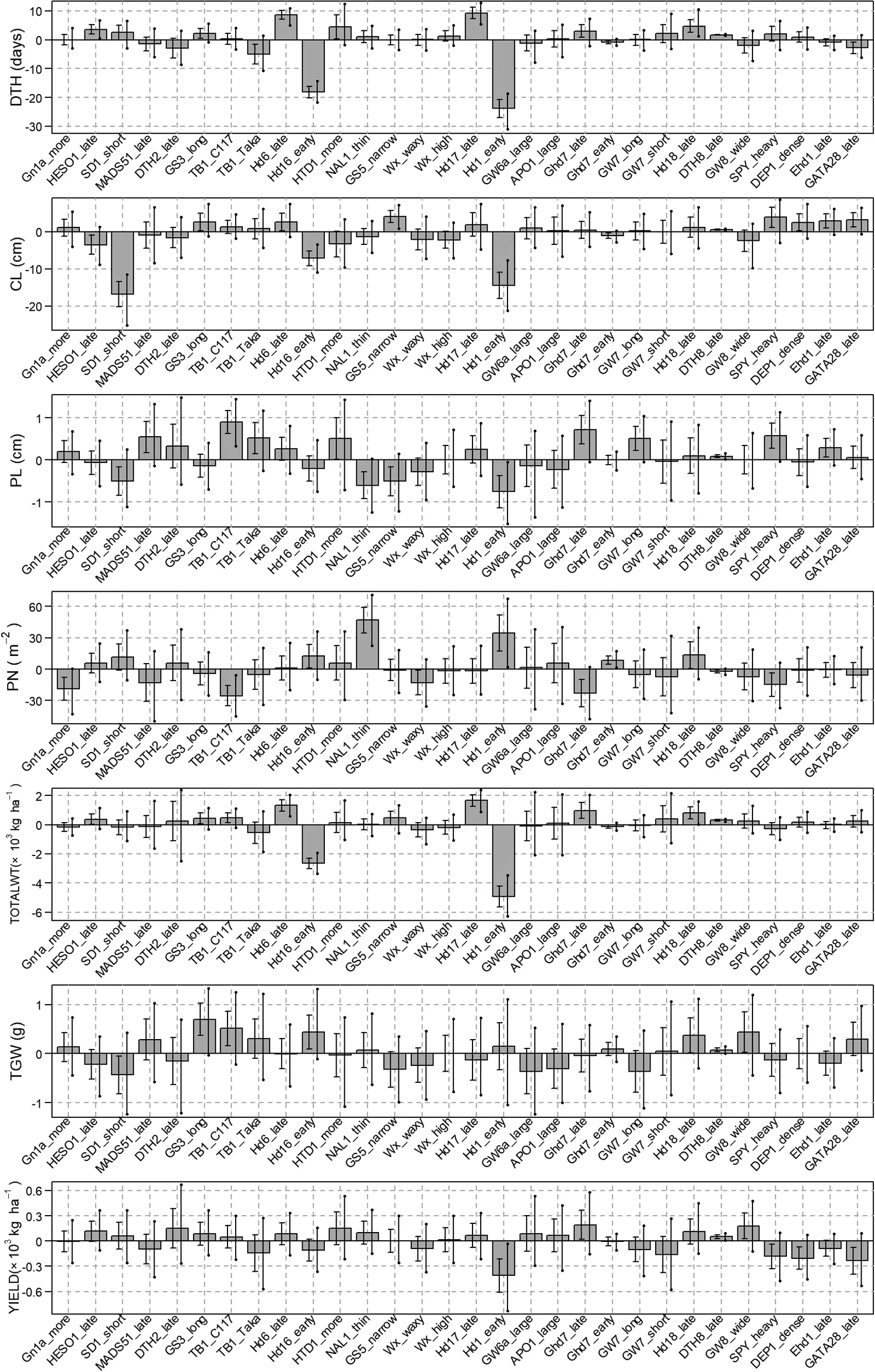
Estimated effects of each functional allele of agronomically important genes on traits. Error bars with horizontal lines indicate standard deviations. Error bars with dots indicate 95% confidence intervals. The effects were estimated using BRR.

**Table 1.** Summary of NARO rice historical phenotype dataset before and after extraction

	Records	Cultivars	Years	Locations	Trials
NARO rice historical phenotype dataset	225,163	5,236	43	111	3,680
Extracted subset	19,218	153	43	81	2,176

**Table 2.** Known functional alleles of the major genes of high-yielding staple rice cultivars released recently

Cultivars	Released year	Estimated effect on YIELD in CV model (×10^3^ kg ha^–1^)	95% credible intervals (×10^3^ kg ha^–1^)	Gn1a_more	SD1_short	TB1_C117	TB1_Taka	HTD1_more	NAL1_thin	Hd1_early	APO1_large	Ghd7_late	Ghd7_early	GW7_long	GW7_short	SPY_heavy	DEP1_dense
K666	2005	+0.46 ± 0.07	+0.34~+0.61	—	✓	—	—	—	—	—	—	—	—	—	—	—	—
B14006	2008	+0.49 ± 0.07	+0.34~+0.62	—	✓	—	—	—	—	—	—	—	—	✓	—	—	✓
K667	2008	+0.71 ± 0.09	+0.53~+0.89	—	—	✓	—	—	✓	—	—	✓	—	✓	—	✓	—
B18016	2009	+0.39 ± 0.09	+0.21~+0.55	—	✓	—	—	—	—	—	—	—	—	—	—	—	—
K679	2010	+0.89 ± 0.11	+0.68~+1.11	—	—	—	—	—	—	—	—	—	—	✓	—	—	—
B18013	2012	+0.10 ± 0.07	–0.04~+0.24	—	✓	—	—	—	—	—	—	—	—	—	—	—	✓
B18022	2013	–0.20 ± 0.06	–0.32~–0.08	—	✓	✓	—	—	—	✓	—	—	—	—	—	✓	—
B17001	2014	+0.57 ± 0.09	+0.39~+0.74	—	—	—	—	—	✓	—	—	✓	—	✓	—	✓	—
B18014	2015	+0.28 ± 0.06	+0.17~+0.39	—	✓	—	—	—	—	—	—	—	—	✓	—	—	—
