# Supplementary material for: Effects of known functional alleles of agronomically important genes on yield-related traits estimated by historical rice breeding data in Japan
Source: Breed Sci. 2026 Jun 11;76(3):229–39. doi: 10.1270/jsbbs.25062 (PMC13709626; doi:10.1270/jsbbs.25062)
Supplement: Supplementary file 1 [file 76_229_s1.pdf]

Chigira et al./Breeding Science
doi: 10.1270/jsbbs.25062

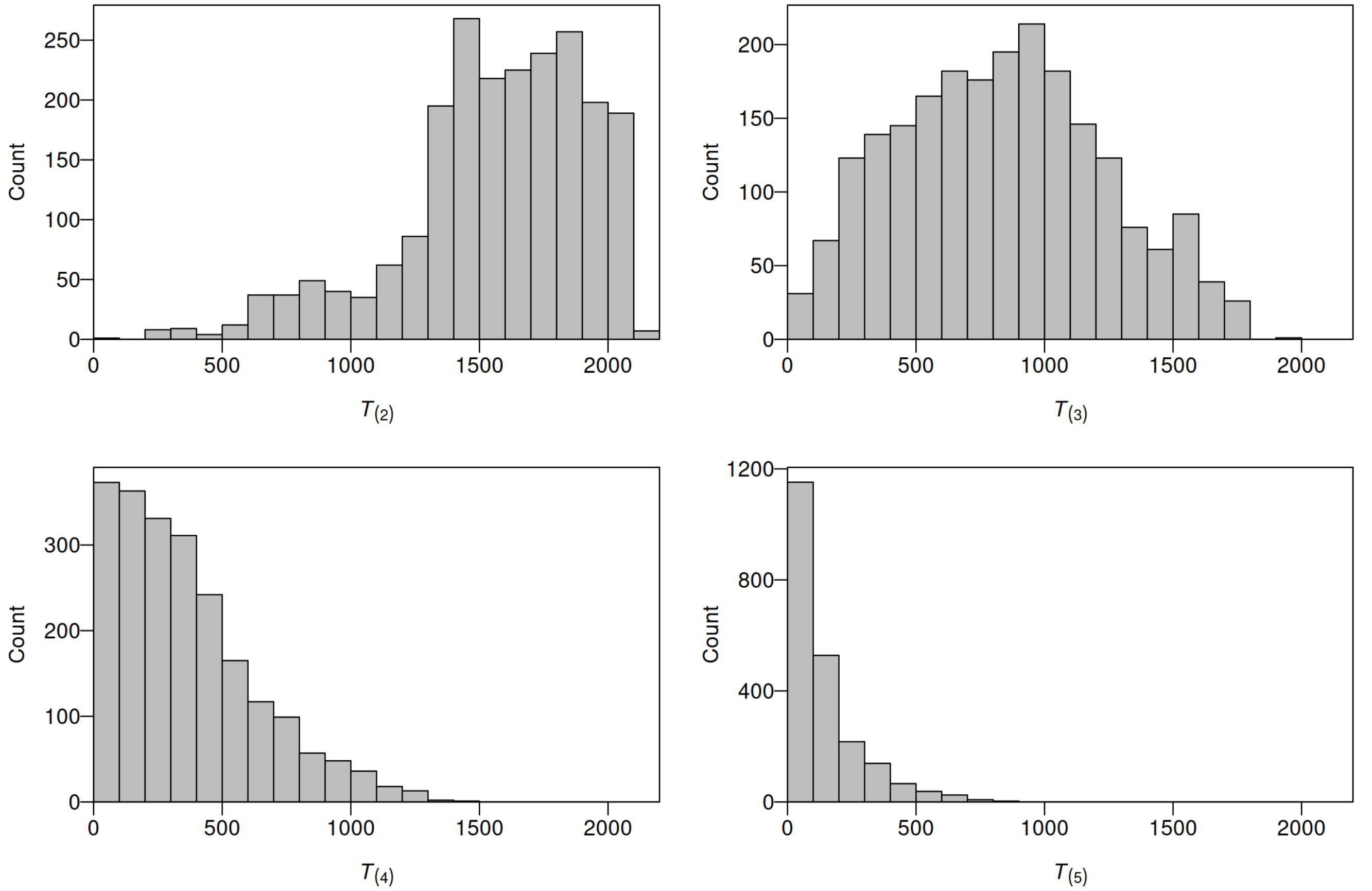

Supplemental Figure 1. Distribution of connectivity indexes for each number of cultivars.

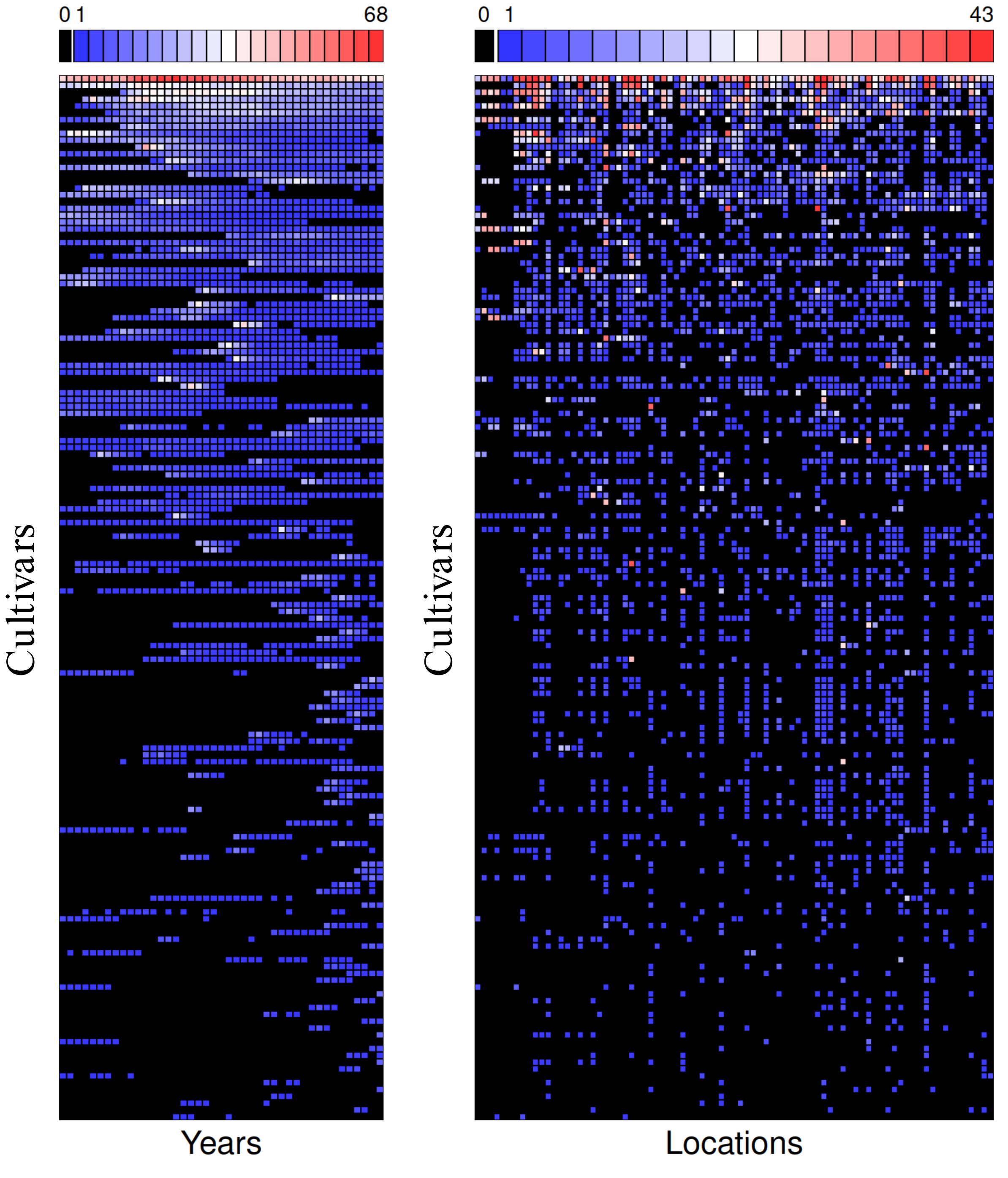

Supplemental Figure 2. Count of available records in each combination of cultivars and years, or cultivars and locations. Cultivars are listed from top to bottom in order of the number of records. The years are listed from left to right, from 1980 to 2022. Locations are listed from left to right in order of high to low latitude.

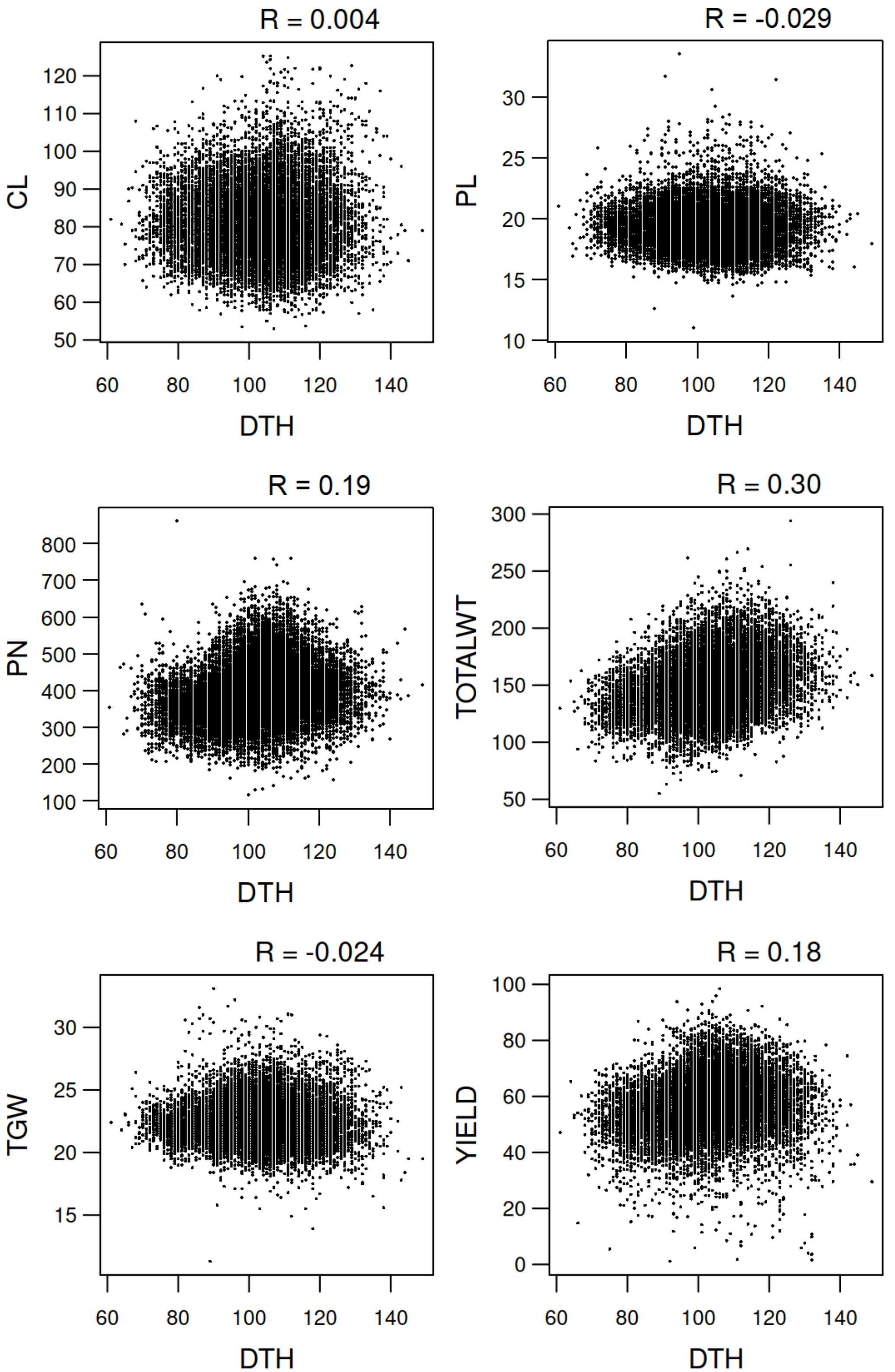

Supplemental Figure 3. Correlation of days to heading and the other traits.

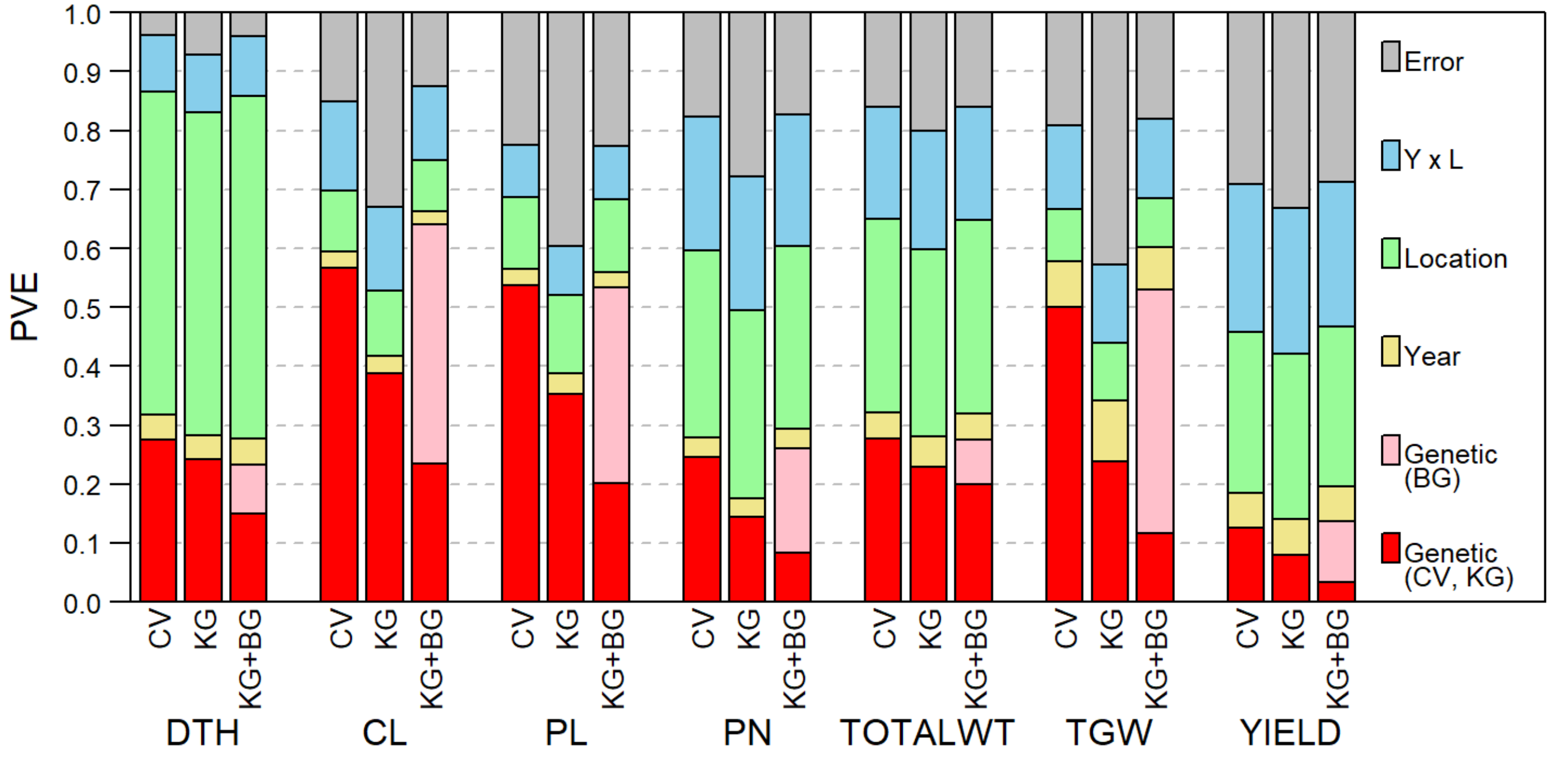

Supplemental Figure 4. Phenotypic variance explained by environmental and genetic factors in each trait using Bayes B regression.
